# Oroxylin A, but Not Vasopressin, Ameliorates Cardiac Dysfunction of Endotoxemic Rats

**DOI:** 10.1155/2012/408187

**Published:** 2012-10-24

**Authors:** Chin-Hung Liu, Mei-Fang Chen, Tzu-Ling Tseng, Lih-Geeng Chen, Jon-Son Kuo, Tony Jer-Fu Lee

**Affiliations:** ^1^Department of Life Sciences, College of Life Sciences, Tzu Chi University, No. 701, Section 3, Chung Yan Road, Hualien, Taiwan; ^2^Center for Vascular Medicine, College of Life Sciences, Tzu Chi University, No. 701, Section 3, Chung Yan Road, Hualien, Taiwan; ^3^Department of Research, Buddhist Tzu Chi General Hospital and Tzu Chi College of Technology, Hualian, Taiwan; ^4^Medical Research, College of Medicine, Tzu Chi University, No. 701, Section 3, Chung Yan Road, Hualien, Taiwan; ^5^Graduate Institute of Biomedical and Biopharmaceutical Sciences, College of Life Sciences, National Chiayi University, Chiayi, Taiwan; ^6^Institute of Pharmacology and Toxicology, College of Medicine, Tzu Chi University, No. 701, Section 3, Chung Yan Road, Hualien, Taiwan; ^7^Department of Pharmacology, Southern Illinois University School of Medicine, Springfield, IL, USA

## Abstract

The mortality in septic patients with myocardial dysfunction is higher than those without it. Beneficial effects of flavonoid oroxylin A (Oro-A) on endotoxemic hearts were evaluated and compared with that of arginine vasopressin (AVP) which is used to reverse hypotension in septic patients. Endotoxemia in rats was induced by one-injection of lipopolysaccharides (LPS, 10 mg/kg, i.p.), and hearts were isolated 5-hrs or 16-hrs later. Isolated hearts with constant-pressure or constant-flow mode were examined by Langendorff technique. Rate and force of contractions of isolated atrial and ventricular strips were examined by tissue myography. Isolated endotoxemic hearts were characterized by decreased or increased coronary flow (CF) in LPS-treated-for-5hr and LPS-treated-for-16-hr groups, respectively, with decreased inotropy in both groups. Oro-A-perfusion ameliorated while AVP-perfusion worsened the decreased CF and inotropy in both preparations. Oro-A and AVP, however, did not affect diminished force or rate of contraction of atrial and ventricular strips of endotoxemic hearts. Oro-A-induced CF increase was not affected following coronary endothelium-denudation with saponin. These results suggest that Oro-A ameliorates LPS-depressed cardiac functions by increasing CF, leading to positive inotropy. In contrast, AVP aggravates cardiac dysfunction by decreasing CF. Oro-A is a potentially useful candidate for treating endotoxemia complicated with myocardial dysfunction.

## 1. Introduction

Sepsis is a systemic response to infection [1], and may lead to septic shock which is one of the primary causes of death in the intensive care unit in many countries [2]. The most common cause of sepsis is an exposure to lipopolysaccharides (LPS), the structural component of a Gram-negative bacterial membrane, and key symptoms may include hypotension [3] and multiple organ failure [4]. During septic shock, myocardial depression also may be present and is characterized by impaired myocardial contractility and reduced ejection fraction [2]. The mortality in septic patients with myocardial dysfunction is higher by 50–70% than those without myocardial dysfunction [5]. Although many factors have been proposed to cause myocardial depression in septic shock [6–8], the complicated pathogenesis of cardiac dysfunction in septic shock remains unclear.

Oroxylin A (Oro-A, 5,7-dihydroxy-6-methoxyflavone), one of the main bioactive component in the root of *Scutellaria baicalensis*, is a conventional herbal medicine widely prescribed as an analgesic, antipyretic, anti-inflammation, anticancer, antiviral and antibacterial infections remedy [9]. It also is an antioxidant that depresses generation of superoxide and nitric oxide (NO) [10]. In our previous report, Oro-A, via inhibition of nuclear factor-kappa B (NF-*κ*B) activation, blocks LPS-induced expressions of inducible nitric oxide synthase (iNOS) and cyclooxygenase (COX)-2 in macrophages [11]. In addition, our preliminary in vivo experimentation demonstrated that Oro-A administered (i.v.) after establishment of LPS-induced septic shock (posttreatment) in anesthetized rats reversed the depressed heart rate (HR) and hypotension to normal ranges in 10 min with significantly improved survival rate. The exact mechanisms of action of Oro-A in ameliorating the myocardial dysfunction and hypotension in endotoxemia, however, are not clarified.

In the present study, we tested the hypothesis that Oro-A posttreatment (administered after establishment of endotoxemia or endotoxemic shock) improved coronary flow (CF) and cardiac function of the endotoxemic rat. Clinically, arginine vasopressin (AVP) is increasingly used to raise the systemic arterial blood pressure in septic patients who are refractory to catecholamine or conventional treatments [12]. AVP, however, has been shown to cause constriction of coronary arteries [13], leading to impairment of cardiac index and systemic oxygen delivery [14, 15]. Accordingly, chronic administration of AVP in patients with cardiac dysfunction should be cautious [16, 17]. We, therefore, evaluated and compared the cardiac effects of AVP and Oro-A. Our results indicated that Oro-A reversed while AVP further decreased the diminished CF and cardiac contractile force in isolated hearts from endotoxemic rats.

## 2. Materials and Methods

### 2.1. Drugs and Chemicals

LPS (derived from *Escherichia coli*, serotype 0127:B8), dimethyl sulfoxide (DMSO), arginine vasopressin (AVP), 5-hydroxytryptamine (5-HT), sodium nitroprusside (SNP), and saponin were obtained from Sigma Chemical (St. Louis, MO, USA). Acetone, chloroform, methanol, and hexane were purchased from Mallinckrodt (St. Louis, MO, USA).

### 2.2. Extraction and Purification of Oro-A from *S. baicalensis*

Oro-A was extracted from dried *S*. *baicalensis* [18]. In brief, dried *S*. *baicalensis *roots were cut into small pieces, which were then immersed and extracted with 10-fold (v/w) acetone at room temperature once every 2 weeks for 2 times. The acetone extracts were subjected to column chromatography on silica gel eluted with chloroform and chloroform-methanol and rechromatographed on silica gel eluted with hexane-acetone to yield Oro-A. The compound was identified by direct comparison of its electrospray ionization (ESI-) mass, ^1^H-, and ^13^C-nuclear magnetic resonance (NMR) spectroscopic data with authentic samples. The purity exceeded 99.5% as determined by high-performance liquid chromatography (HPLC). Because of the low water solubility, Oro-A was dissolved in 100% DMSO as a 100 mM stock solution and then added to the protein-free Krebs-Henseleit (K-H) buffer to make 10 or 20 *μ*M Oro-A solution before use. The K-H buffer contains (in mmol/L) NaCl 118, KCl 4.8, CaCl_2_ 1.3, MgSO_4_ 1.2, NaHCO_3_ 25, KH_2_PO_4_ 1.2, and glucose 11, which was filtered through a 0.22 *μ*m filter disk (Millipore, Eschborn, Germany) before use.

### 2.3. Animal Models

All animal protocols were approved by the Animal Care and Use Committee of Tzu-Chi University. Adult male Sprague-Dawley rats (280–350 g), purchased from the BioLASCO Co., Ltd. Taipei, Taiwan, were housed in the Tzu-Chi University's animal quarters under a 12 hrs light/dark cycle. All rats were fed with a standard ration and tap water ad libitum. Endotoxemia was induced in conscious rats by injection of LPS (10 mg/kg in 1 mL of saline, i.p.) [19], while control group by injection of saline (1 mL/kg, i.p.).

**Figure 1:**
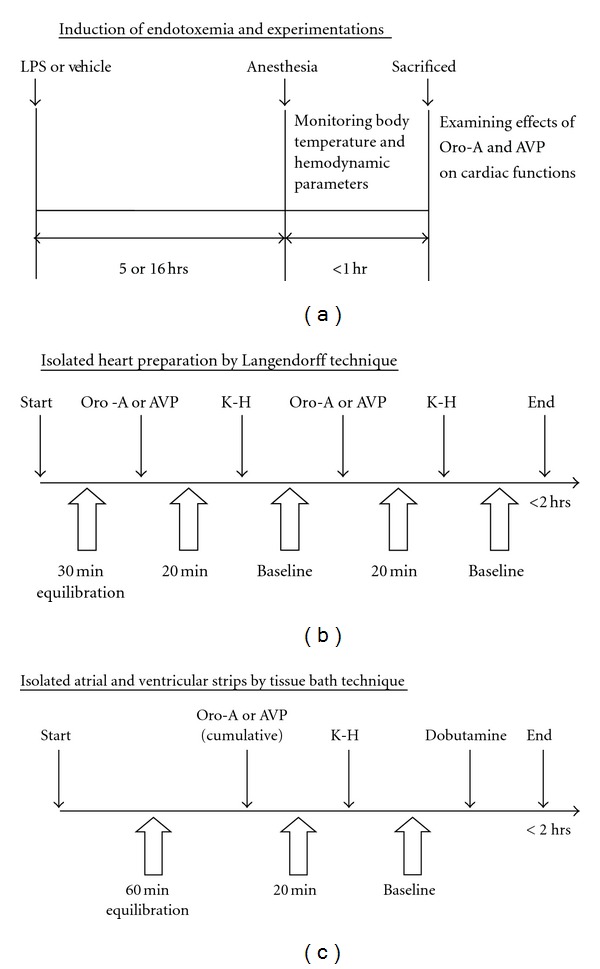
Experimental designs. Time schedules for LPS induction of endotoxemia in rats are shown in panel (a). Time schedules for studying effects of Oro-A and AVP on isolated endotoxemic heart are shown in panel (b), and on isolated atrial and ventricular strips in panel (c). LPS (lipopolysaccharide); K-H (Krebs-Henseleit solution); Oro-A (oroxylin A); AVP (arginine vasopressin).

Sixty-five rats were divided into three groups. (I) The control group: rats were treated with saline (1 mL/kg, i.p.) and sacrificed 5 hrs later. (II) The LPS-treated-for-5 hr endotoxemic group: rats were treated with LPS (10 mg/kg, i.p.) and sacrificed 5 hrs later. (III) The LPS-treated-for-16 hr endotoxemic shock group: rats were treated with LPS (10 mg/kg, i.p.) and sacrificed 16 hrs later. Five hrs or 16 hrs after saline or LPS treatment, rats were anesthetized by sodium pentobarbital (50 mg/kg, i.p.) and the mean arterial pressure (MAP, mmHg), HR, and body temperature (BT, °C) were measured. Animals were then heparinized (1000 IU/kg, i.p.) for 15 min before sacrifice (Figure 1(a)). Hearts were removed and prepared for Langendorff studies, and atrial and ventricular strips were dissected from some hearts for tissue bath studies (see below).

The rectal temperature, as an index of steady core body temperature, was recorded with a thermocouple probe coupled with a 7000 H microcomputer thermometer (Jenco Electronics Ltd., Taipei, Taiwan). The probe lubricated with Vaseline was inserted 5 cm into the rectum to ensure a reliable measurement. The femoral artery was cannulated with a polyethylene-50 (PE-50) catheter connected to a pressure transducer (P231D, Statham, Oxnard, CA, USA) for measuring the MAP and HR [20], which were displayed on a MP35 polygraph recorder (Biopac System, Inc., Santa Barbara, CA, USA).

### 2.4. Choice of Experimental Dosage of Oro-A and AVP

The choice of 10 and 20 *μ*M of Oro-A in the present study was based on our previous biochemical studies using cell lines [11] that 17 *μ*M Oro-A transcriptionally inhibited the expression of iNOS and COX-2 induced by LPS. Concentrations of AVP of 0.2 and 0.4 IU/L (equivalent to 0.003 and 0.006 IU/min calculated as 1 international unit = 4 nM AVP and based on 14 mL/min of perfusion flow rate) used in the present studies are comparable to those used by other investigators [21, 22]. The clinical doses used in the human adults are infused at a rate of 0.01 to 0.04 IU/min [23].

### 2.5. Langendorff Preparations

The cardiac functions were determined by a modified isovolumetric Langendorff technique [24]. After thoracotomy under anesthesia as described above, hearts were quickly excised and placed in 4°C K-H buffer. A fluid-filled latex balloon, inserted into the left ventricle (LV) cavity via the mitral valve, was linked to a transducer connected with MP35 polygraph recorder for measuring left ventricular systolic pressure (LVSP) and HR. The balloon was inflated after insertion to reach a left ventricular end-diastolic pressure (LVEDP) of 5 to 10 mmHg, which remained unchanged throughout the experiment. Cardiac functions were evaluated upon left ventricular developed pressure (LVDP) calculated as the difference between LVSP and LVEDP, and the rate-pressure product (RPP, indicative of cardiac work) [25] was calculated as the product of LVDP and HR [26].

The constant pressure heart preparation was that the coronary perfusion pressure (CPP) was maintained constant at 96 cm H_2_O monitored by a pressure transducer with MP35 polygraph recorder. This allowed to measure the CF by collecting the effluent dripping from the heart [24]. Thus, an increase or decrease of the CF represents dilatation or constriction, respectively, of the coronary artery. On the other hand, in the constant flow heart preparation, the CF was maintained constant at 14 mL/min. This allowed to measure the CPP (indicative of coronary resistance). An increase or decrease of the CPP indicates constriction or dilatation of the coronary, respectively [27].

The isolated heart was perfused through an aortic cannula with oxygenated K-H buffer (95% O_2_ + 5% CO_2_, pH 7.4) containing 0.02% (v/v) DMSO (the maximum final concentration of used as Oro-A in K-H buffer). Perfusion fluid and bath temperature were maintained at 37°C by a thermostatically controlled water circulator. After 30 min of baseline equilibration, the heart received Oro-A (10 and 20 *μ*M) or AVP (0.2 and 0.4 IU/L) in randomized order, each for 15 min to achieve a steady state. After 20 min washout, Oro-A or AVP was administered (Figure 1(b)). All measurements were taken during the last 5 min of each experimental period.

### 2.6. Coronary Endothelium Denudation

In order to assess possible role of endothelium in coronary vasomotor effect of Oro-A, vasomotor function of the coronary with or without the endothelium was evaluated. After 30 min of baseline equilibration in the Langendorff technique with constant flow heart preparation, 1 *μ*M 5-HT (submaximum concentration) was perfused to induce endothelium-dependent coronary dilatation [28] as indicated by a decrease in the CPP. The hearts were then perfused with saponin (50 *μ*g/mL in the K-H buffer) 3 times (5 min of saponin followed by 5 min K-H buffer each time) to ablate endothelium [28], then the heart received Oro-A (10 *μ*M) and 5-HT (1 *μ*M). A complete denudation was indicated by lack of 5-HT-induced vasodilation or decreased CPP. At the end of the experiment, 100 *μ*M SNP was perfused to induce maximum percent of relaxation of coronary arteries, and drug-induced relaxation was estimated as percent of that induced by SNP. All measurements were taken during the last 5 min of each experimental period.

### 2.7. Force and Rate of Contraction of Isolated Atria and Ventricular Strips

In order to further confirm whether Oro-A and AVP exhibit direct myocardial effect, beat rate (right atrium) and isometric forces of isolated heart strips were evaluated. The right atrial and ventricular strips were dissected from the isolated heart and were suspended vertically in a 10 mL tissue bath filled with oxygenated K-H buffer (with 0.02% v/v DMSO) at 35°C [29]. The right atrial strips were allowed to beat spontaneously, and the ventricular strips were contracted by electrical stimulation with a Grass SD-9 stimulator (Grass-Telefactor, RI, USA) at the frequency of 2 Hz, 5 ms of duration, twin pulses and supramaximal (threshold + 25%) voltage [30]. The resting force of both atrial and ventricular strips was adjusted at 0.5 g. Changes in beat rate and isometric force were recorded via a transducer bridge amplifier connected to a MP35 polygraph recorder and stored in a public computer.

After 60 min of baseline equilibration, each preparation was treated with different concentrations of Oro-A (10, 20 *μ*M) or AVP (0.2, 0.4 IU/L) for 10 min each. At the end of each experiment, a drug-free K-H buffer was replaced for 20 min, allowing a recovery to the baseline condition. Dobutamine (1 *μ*M, a *β*_1_-adrenoceptor agonist) then was applied and results served as positive control for atrial rate (AR) and isometric force (Figure 1(c)). The force was calculated as follows Force mg/mg = isometric force mg/preparation wet weight mg [31]. All measurements were taken during the last 5 min of each experimental period.

### 2.8. Statistical Analysis

Data are expressed as means ± SEM. One-way analysis of variance (ANOVA) and Student's *t-*test were employed for comparison within groups the effects of Oro-A, AVP, or 5-HT on their cardiac functions, or coronary vasomotor activities. The differences among groups were compared changes in hemodynamics, cardiac functions or coronary vasomotor activities by two-way ANOVA. Post-hoc analysis was done with SPSS version 13.0 (SPSS Inc., Chicago, IL). Statistical significance was set at *P* < 0.05.

## 3. Results

### 3.1. Changes in Hemodynamics in LPS-Treated Rats

Five hrs and 16 hrs after LPS treatment, rats developed endotoxemia and endotoxemic shock, respectively, characterized by the presence of lassitude, pilo-erection, fever, and tachycardia (Table 1). The MAP remained unchanged 5 hr after LPS treatment (endotoxemia without shock, or early-stage endotoxemia), but decreased by 35% 16 hr after LPS treatment (endotoxemia with shock, or late-stage endotoxemia). None of the rats died in 5 hr after LPS treatment (or LPS-treated-for- 5 hr group), while 2 of 20 (10%) rats died between 8 and 12 hrs after LPS in LPS-treated-for-16 hr group.

### 3.2. Cardiac Dysfunction Induced by LPS

Compared with that of the normal control (Table 1), the CF in constant pressure heart preparation of the LPS-treated-for-5 hr rats was decreased significantly by 45%, while the CPP in constant flow heart preparation was increased significantly by 30%. The CF in the hearts of the LPS-treated-for-16 hr rats, however, was increased significantly by 20%, while the CPP was decreased significantly by 43% (Table 1). From the rats treated with LPS for 5 hrs or 16 hrs, the LVDP and RPP of the isolated hearts were significantly decreased in heart preparations of the constant pressure by 46% and 48%, respectively, and of the constant flow by 52% and 50%, respectively (Table 1). The HR was not significantly changed except a slight but significant (16%) increase in the constant pressure heart preparation from LPS-treated-for-16 hr rats.

Furthermore, contractile force of the isolated right atrial strips (atrial force/AF) from both 5 hr and 16 hr after LPS-treated rats and that of the ventricular strips (ventricular force/VF) from 5 hr after LPS-treated rats were significantly decreased (Table 1). On the other hand, the spontaneous contraction rate of atrial strips (atrial rate/AR) from both 5 hr and 16 hr post-LPS rats were slightly but significantly increased (Table 1), while that of the ventricular rate (VR) was not altered 5 hrs after LPS treatment (LPS-treated-for-5 hr rats).

### 3.3. Effects of Oro-A and AVP on LPS-Induced Endotoxemic Hearts

**Figure 2:**
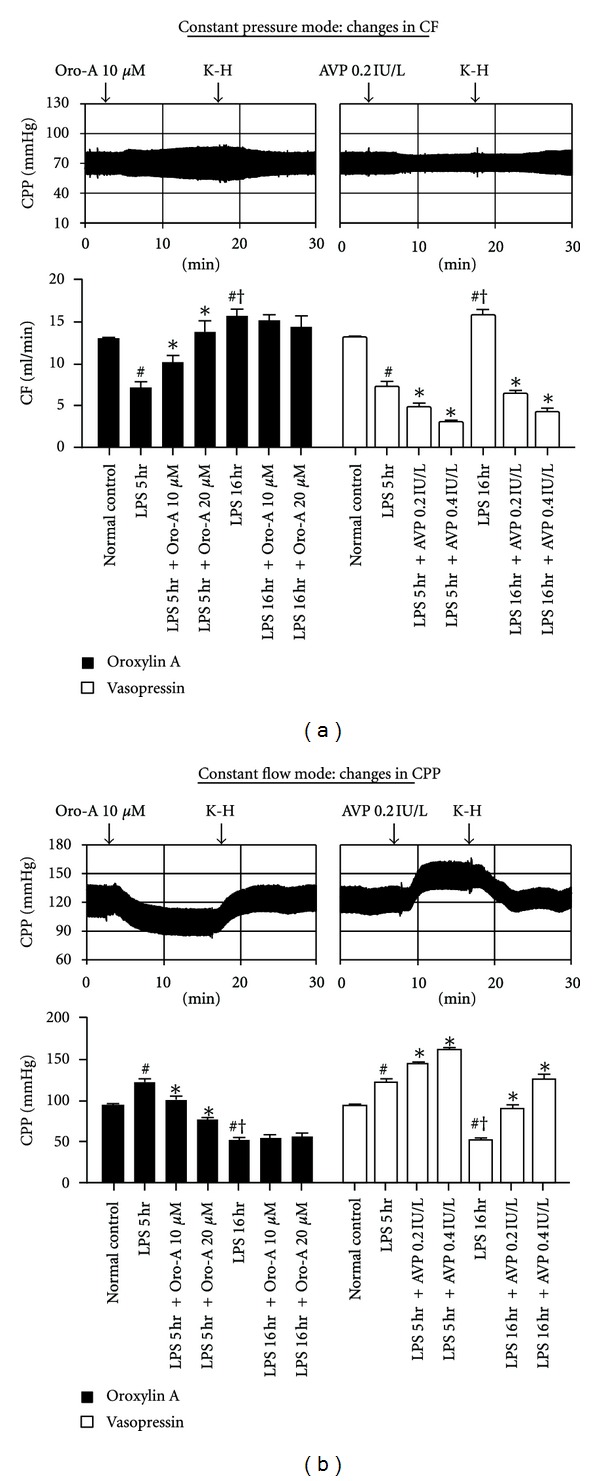
Effects of Oro-A and AVP on the CPP, CF, and coronary arterial function of isolated hearts from endotoxemic rats. The representative tracings in (a) indicate that Oro-A (10 *μ*M) or AVP (0.2 IU/L) perfusion does not affect the mean of CPP; however, Oro-A (10 *μ*M) perfusion increases while AVP (0.2 IU/L) perfusion decreases the pulse of CPP in constant pressure heart preparation from 5 hr post-LPS rats. In constant flow heart preparation (b), Oro-A (10 *μ*M) decreases while AVP (0.2 IU/L) increases the CPP in the constant flow (14 mL/min) heart preparation from 5 hr post-LPS rats. The bar chart in (a) summarize effects of Oro-A (10 and 20 *μ*M, solid bar) and AVP (0.2 and 0.4 IU/L, open bar) on CF in constant pressure heart preparation from 5 hr or 16 hr post-LPS rats. The bar chart in (b) summarize effects of Oro-A (10 and 20 *μ*M, solid bar) and AVP (0.2 and 0.4 IU/L, open bar) on CPP in constant flow heart preparation from 5 hr or 16 hr post-LPS rats. Values of normal control and 5 hr and 16 hr post-LPS rats are from Table 1. Values are mean ± SEM (*n* = 6 each group). *P < 0.05 versus respective LPS groups; ^#^ *P* < 0.05 versus normal control; ^†^ *P* < 0.05 versus 5 hr post-LPS group. CF (coronary flow); CPP (coronary perfusion pressure).

In constant pressure heart preparation from LPS-treated-for-5 hr rats (Figure 2(a), solid bar, *n* = 6 each), Oro-A (10 and 20 *μ*M) concentration-dependently increased CF. Similar concentrations of Oro-A, however, did not affect the already-increased CF in the hearts from LPS-treated-for-16 hr rats. In contrast, AVP (0.2 and 0.4 IU/L) concentration dependently decreased the CF in the hearts of both LPS-treated for 5 hr and 16 hr rats (Figure 2(a), open bar).

In constant flow heart preparation from LPS-treated-for-5 hr rats, Oro-A (10 and 20 *μ*M) in concentration-dependent manner decreased the CPP (Figure 2(b), solid bar). Oro-A at the same concentrations, however, did not affect the already decreased CPP in the hearts of LPS-treated-for-16 hr rats. In contrast, AVP (0.2 and 0.4 IU/L) concentration dependently increased the CPP in the constant flow heart preparation from both LPS-treated-for-5 hr and 16 hr rats (Figure 2(b), open bar).

**Figure 3:**
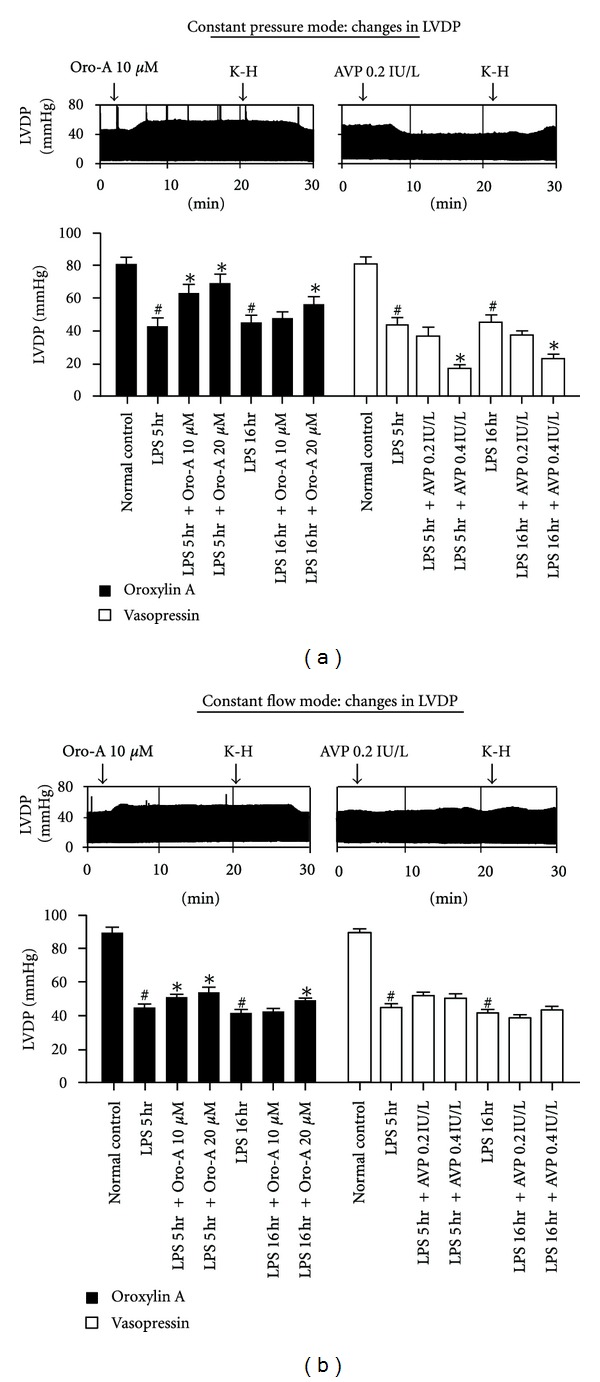
Effects of Oro-A and AVP on the LVDP of isolated hearts from endotoxemic rats. The representative tracings in (a) indicate that Oro-A (10 *μ*M) perfusion increases while AVP (0.2 IU/L) perfusion decreases the LVDP in constant pressure heart preparation from 5 hr post-LPS rats. In constant flow heart preparation (b), Oro-A (10 *μ*M) slightly but significantly increases LVDP, while AVP (0.2 IU/L) is without effect. The bar chart in (a) summarizes effects of Oro-A (10 and 20 *μ*M, solid bar) and AVP (0.2 and 0.4 IU/L, open bar) on LVDP in constant pressure heart preparation from 5 hr or 16 hr post-LPS rats. The bar chart in (b) summarize effects of Oro-A (10 and 20 *μ*M, solid bar) and AVP (0.2 and 0.4 IU/L, open bar) on LVDP in constant flow heart preparation from 5 hr or 16 hr post-LPS rats. Values of normal control, and 5 hr and 16 hr post-LPS rats are from Table 1. Values are mean ± SEM (*n* = 6 each group). *P < 0.05 versus respective LPS groups; ^#^ *P* < 0.05 versus normal control. LVDP (left ventricular developed pressure).

The diminished LVDP of the hearts from LPS-treated for 5 hr and 16 hr rats was reversed by Oro-A, but was further decreased by AVP in constant pressure heart preparation (Figure 3(a)). In constant flow heart preparations from LPS-treated-for-5 hr and 16 hr rats, Oro-A also slightly but significantly increased the LVDP, while AVP was without any effect (Figure 3(b)).

**Figure 4:**
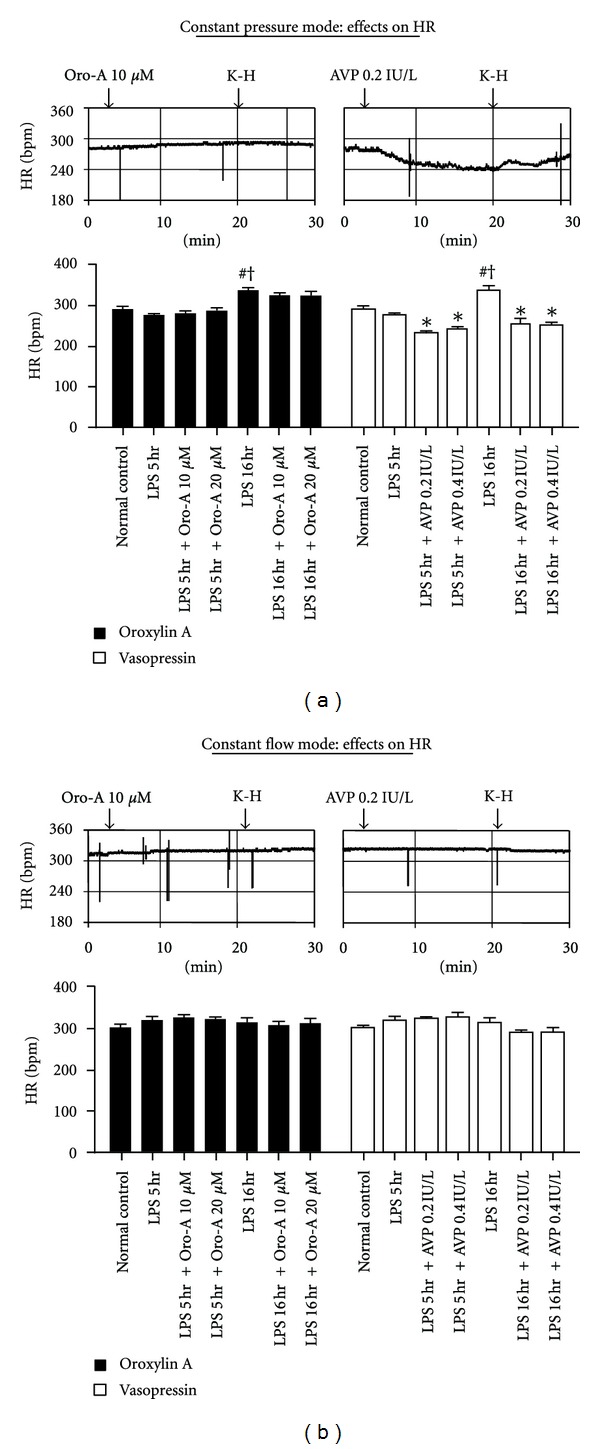
Effects of Oro-A and AVP on the HR of isolated hearts from endotoxemic rats. The representative tracings in (a) and (b) indicate that Oro-A (10 *μ*M) perfusion does not affect the HR in either constant pressure or constant flow heart preparation from 5 hr post-LPS rats. AVP (0.2 IU/L) perfusion, however, decreases HR in constant pressure heart preparation but does not affect that in constant flow heart preparation. The bar chart in (a) summarizes effects of Oro-A (10 and 20 *μ*M, solid bar) and AVP (0.2 and 0.4 IU/L, open bar) on HR in constant pressure heart preparation from 5 hr or 16 hr post-LPS rats. The bar chart in (b) summarizes effects of Oro-A (10 and 20 *μ*M, solid bar) and AVP (0.2 and 0.4 IU/L, open bar) on HR in constant flow heart preparation from 5 hr or 16 hr post-LPS rats. Values of normal control, and 5 hr and 16 hr post-LPS rats are from Table 1. Values are mean ± SEM (*n* = 6 each group). *P < 0.05 versus respective LPS groups; ^#^ *P* < 0.05 versus normal control; ^†^ *P* < 0.05 versus 5 hr post-LPS group. HR (heart rate).

Furthermore, Oro-A perfusion did not affect the HR in either constant pressure (Figure 4(a), solid bar) or constant flow (Figure 4(b), open bar) heart preparations of LPS-treated-for-5 hr and 16 hr rats. In contrast, AVP (0.2 and 0.4 IU/L) perfusion decreased HR in constant pressure (Figure 4(a), solid bar) heart preparation of LPS-treated-for-5 hr and 16 hr rats, but did not affect that in constant flow heart preparations (Figure 4(b), open bar).

**Figure 5:**
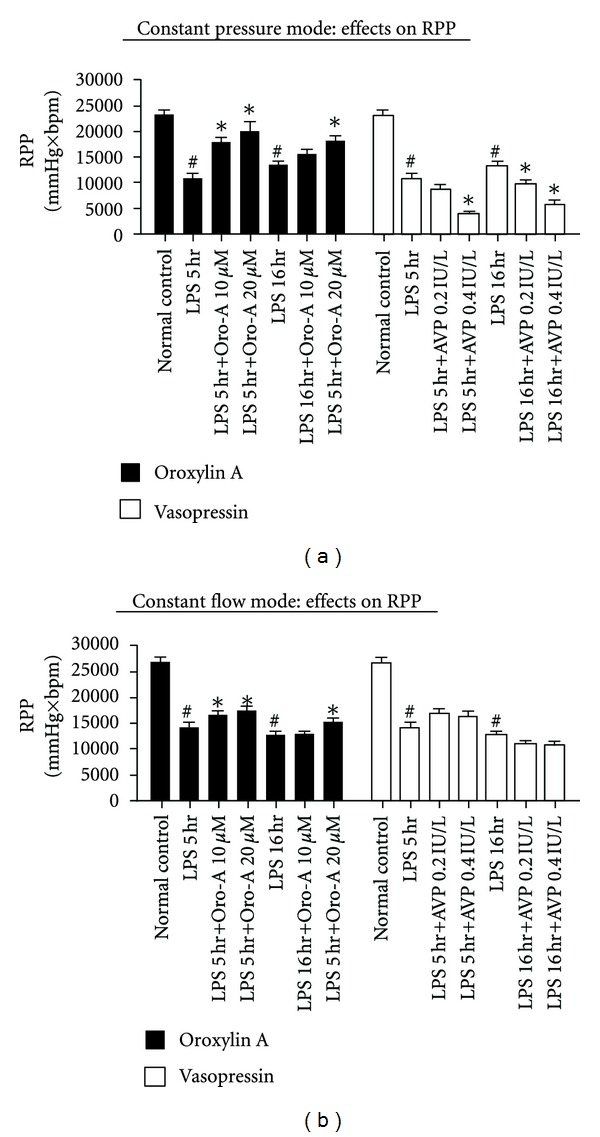
Effects of Oro-A and AVP on the RPP of isolated hearts from endotoxemic rats. (a) summarizes effects of Oro-A (10 and 20 *μ*M, solid bar) and AVP (0.2 and 0.4 IU/L, open bar) on RPP in constant pressure heart preparation from 5 hr or 16 hr post-LPS rats. (b) summarizes effects of Oro-A (solid bar) and AVP (open bar) on RPP in constant flow heart preparation from 5 hr or 16 hr post-LPS rats. Values of normal control and 5 hr and 16 hr post-LPS rats are from Table 1. Values are mean ± SEM (*n* = 6 each group). *P < 0.05 versus respective LPS groups; ^#^ *P* < 0.05 versus normal control. RPP (rate-pressure product).

In isolated hearts of constant-pressure and constant-flow heart preparations from LPS-treated-for-5 hr and 16 hr rats, Oro-A perfusion increased RPP (Figures 5(a) and 5(b), solid bar, *n* = 6 each). In contrast, AVP significantly reduced RPP in the constant pressure heart preparation but did not significantly affect those in the constant flow heart preparations (Figures 5(a) and 5(b), open bar, *n* = 6 each).

### 3.4. Oro-A-Induced Decrease of Coronary Perfusion Pressure (CPP) is Independent of the Endothelium

**Figure 6:**
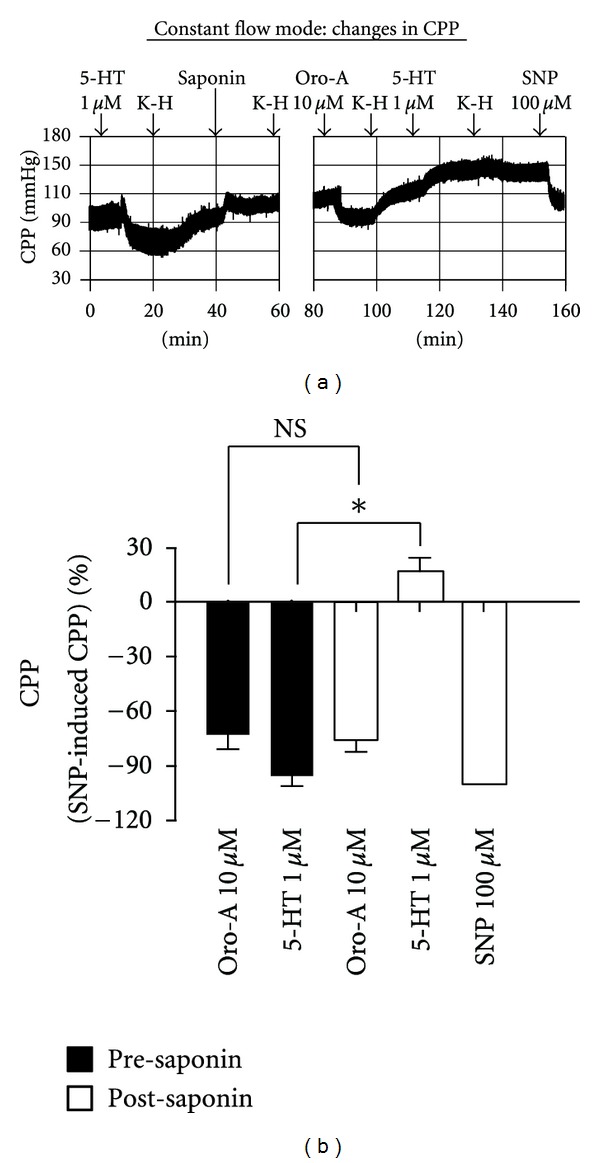
Effects of endothelium-denudation on CPP induced by 5-HT and Oro-A in isolated hearts from normal control rats. A representative tracing in (a) shows that 5-HT (1 *μ*M) decreased CPP. After intravascular infusion with saponin (50 *μ*g/mL) for 15 min, the decreased CPP induced by Oro-A (10 *μ*M) was not affected, while that induced by 5-HT was blocked and converted to an increase. These effects of Oro-A (10 *μ*M) and 5-HT (1 *μ*M) on CPP (percentage of 100 *μ*M SNP-induced) before (solid bar) and after (open bar) saponin are summarized in (b). Values are mean ± SEM (*n* = 4). *P < 0.05 versus presaponin; NS (not significant, *P* > 0.05). 5-HT (5-hydroxytryptamine); SNP (sodium nitroprusside).

In constant flow heart preparation from normal rats, 5-HT (1 *μ*M) and Oro-A (10 *μ*M) decreased the CPP indicative of decreased coronary resistance. After endothelium denudation by saponin (50 *μ*g/mL), the decrease of CCP induced by Oro-A was not significantly affected. The decrease induced by 5-HT, however, was converted to increase (Figure 6, *n* = 4).

### 3.5. Failure of Oro-A and AVP to Affect Force or Rate of Isolated Atrial and Ventricular Strips from Endotoxemic Hearts

**Figure 7:**
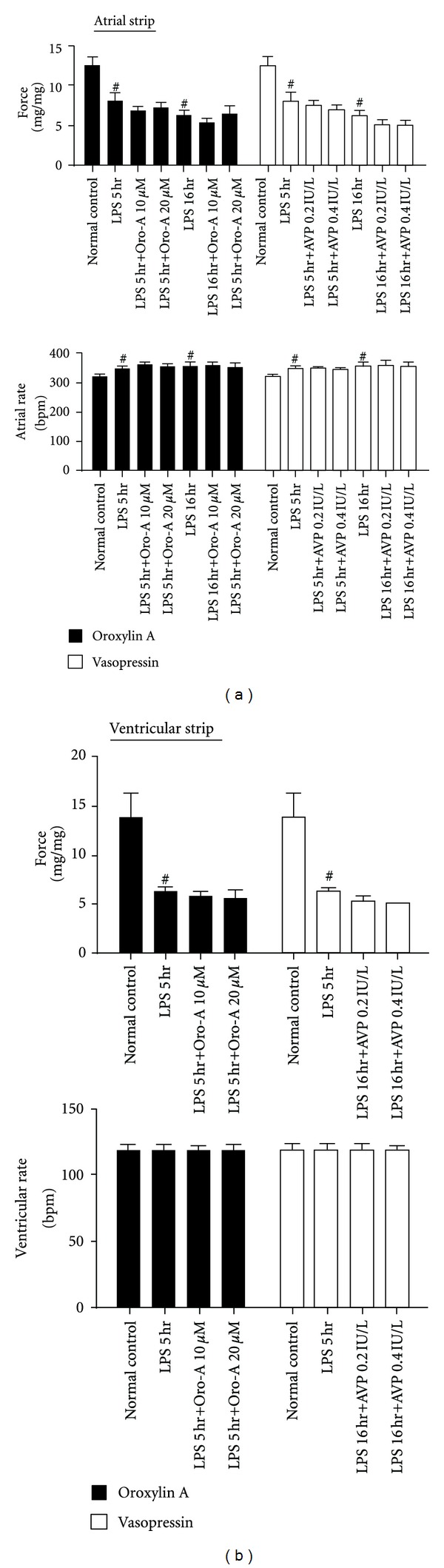
Effects of Oro-A and AVP on force and rate of isolated atrial and ventricular strips from endotoxemic rats. (a) summarizes effects of Oro-A (10 and 20 *μ*M; solid bars) and AVP (0.2 and 0.4 IU/L; open bars) on force and rate of the isolated atrial strips from 5 hr and 16 hr post-LPS rats. (b) summarize effects of Oro-A (10 and 20 *μ*M; solid bar) and AVP (0.2 and 0.4 IU/L; open bars) on electrically-paced force and rate of the ventricular strips from 5 hr post-LPS rats. Values of normal control, and 5 hr or 16 hr post-LPS rats are from Table 1. Values are mean ± SEM (*n* = 5 ~ 6 each group). ^#^ *P* < 0.05 versus normal control.

The spontaneous contractile force of atrial strips from both LPS-treated-for-5 hr and 16 hr rats and the electrically-paced force of the ventricular strips from LPS-treated-for-5 hr rats were significantly decreased comparing to those of the respective controls (Figures 7(a) and 7(b)). On the other hand, the atrial beat rate of both LPS-treated-for-5 hr and 16 hr rats was slightly but significantly enhanced, while the ventricular beat rate of LPS-treated-for-5 hr rats remained unaltered. Oro-A (10 and 20 *μ*M) and AVP (0.2 and 0.4 IU/L) did not affect the atrial force (Figure 7(a), *n* = 6 each) or atrial rate (Figure 7(a), *n* = 6 each) of the isolated atrial strips from both LPS-treated rats. Similarly, Oro-A and AVP at similar concentrations did not affect the electrically-paced force or beat rate of the ventricular strips from LPS-treated-for-5 hr rats either (Figure 7(b), *n* = 5 each).

## 4. Discussion and Conclusion

In the present study, we demonstrated that Oro-A ameliorated while AVP aggravated the LPS-depressed cardiac function in the early-stage (5 hrs after LPS treatment) and late-stage (16 hrs after LPS treatment) endotoxemia by monitoring CF and cardiac inotropy. We reported here also for the first time that Oro-A induced endothelium-independent coronary vasodilation of the isolated hearts. Furthermore, the decreased CF in isolated hearts from the early-stage endotoxemic rats was reversed significantly by Oro-A which, however, did not affect the already increased CF in isolated hearts of the late-stage endotoxemia. In addition, diminished LVDP (indicative of cardiac contractility) and RPP (indicative of cardiac work) of the isolated hearts from both early- and late-stage endotoxemic rats were significantly reversed toward normal ranges by Oro-A without significant effect on the altered HR. The increased CF induced by Oro-A is expected to improve myocardial blood flow and oxygen supplies. No change of the HR by Oro-A suggests additional benefit of no further increase in myocardial oxygen demands. In contrast, AVP further decreased CF, LVDP, and RPP in isolated hearts of both early- and late-stage endotoxemic rats, suggesting its potential detrimental effects on the endotoxemic heart.

The findings of decreased LVDP, right atrial, and right ventricular forces in LPS-induced endotoxemic rats in the present study are in line with the results found in septic patients and other different animal models of sepsis [2, 32]. LPS treatment caused bimodal effects on the CF. In the early-stage endotoxemia, the CF was decreased with increased CPP and coronary microvascular resistance. Similar results have been reported by others [24, 33–35]. This coronary vasoconstriction has been suggested to involve release of vasoconstrictors including endothelin-1 (ET-1, a potent vasoconstrictor and proinflammatory peptide) [36] from the coronary and endocardial endothelial cells and/or impaired vasodilator responses of NO [33]. Therefore, the increased coronary resistance in early-stage endotoxemic hearts may lead to loss of local regulatory mechanism and an increased propensity for coronary vasospasm, myocardial ischemia and coronary dysfunction [37]. On the other hand, in the late-stage endotoxemia, the CF was increased with decreased CPP and coronary microvascular resistance. The coronary vessel already relaxed considerably and, therefore, Oro-A did not cause any further relaxation beyond the capacity of the vessels. The increased coronary vasodilation in the late-stage endotoxemia is likely due to increased expression of iNOS, which produces high levels of NO, in the myocardium [19]. Thus, coronary vascular tone increases in the early-stage and decreases in the late-stage endotoxemia. This difference in hemodynamic changes in endotoxemia may be determined by the balance between available vasoconstrictors such as ET-1 and dilators such as NO at different stages after endotoxin contamination. 

The present results indicated that Oro-A had greater improvement on LVDP and RPP at early-stage than that at late-stage endotoxemia. One logical explanation for this difference is that both CF-related and CF-unrelated mechanisms are involved in positive inotropic effects of Oro-A, and that a smaller CF-related effect than CF-unrelated effect of Oro-A in found at late-stage endotoxemia. This is expected since the coronary vasodilation may already be almost maximal at the late-stage endotoxemia, and therefore may not dilate significantly further or affect the already increased CF in the hearts. These results favor the idea that Oro-A improvement of LVDP and RPP is CF-related in the early-endotoxemic period. It is possible that the CF-unrelated positive inotropic effect may become more determinant in the late-endotoxemic period. The exact mechanisms underlying the CF-unrelated positive inotropic effect of Oro-A remain unknown. It may be due to Oro-A reduction of oxidative stress or removal of free radicals [10, 38] in endotoxemia.

The decreased LVDP and HR induced by AVP administration was found only in the constant-pressure heart preparations, suggesting that altered inotropic and chronotropic effects by AVP also depend on the CF-related mechanisms. Our findings are consistent with those reported by others [15, 21] that AVP-induced decrease of myocardial contractility is most likely due to a decreased CF. However, there was a lack of correlation between CF and corresponding decrease in LVDP and RPP induced by 0.2, U/L AVP in the early-stage endotoxemia, although positive correlation between CF and RPP in the late-stage was found (Figure 5(a)). The exact reason for the difference in findings in different stages is not known. It may be due to that in the late-stage endotoxemia, the coronary vessel relaxed considerably more and, therefore, AVP induced greater vasoconstrictor response. These results suggest that CF-related mechanisms of AVP are more significant in the late-stage than the early-stage endotoxemia, an interesting finding different from that found for Oro-A effects. Furthermore, positive correlations between CF and corresponding decrease in LVDP and RPP induced by 0.4, U/L AVP in both early- and late-stage were found. It appears that the consistent negative inotropic effects of AVP may be induced more likely by its dose higher than 0.2, U/L (equivalent to 0.003 U/min) in the early-stage endotoxemia. These results further favor the coronary vascular effects of Oro-A and AVP in modulating cardiac functions. This information may be helpful in managing endotoxemia, particularly, in those with cardiac dysfunction.

The greater effects Oro-A and AVP on changing LVDP and RPP in the constant-pressure (Figures 3(a) and 5(a)) than those in the constant-flow (Figures 3(b) and 5(b)) heart preparations are consistent with the explanation by the reported “Gregg”s phenomenon' [39] or the “garden-hose effect” [40], in which the increase of heart contractility was a result of elevated CF in the constant-pressure heart preparations. 

Oro-A-induced coronary relaxation with increased CF is endothelium independent (Figure 6). Since endotoxemia may render coronary endothelial dysfunction [41], endothelium-independent vasodilation induced by Oro-A, therefore, becomes an interesting and clinically important mechanism. This is supported by the report that Oro-A represses the phorbol-12-myristate-13-acetate (PMA, a protein kinase C/PKC activator)-induced translocation of PKC-*δ* [42] which is present in the vascular smooth muscle cells [43]. Incidently, physiological concentrations of AVP, which constricts vascular smooth muscle by directly acting on V_1a_ receptors [44] leading to activation of PKC [45]. These results provide additional evidence justifying the different vascular responses induced by Oro-A and AVP.

Furthermore, an increased HR was observed in the constant-pressure, but not the constant-flow, heart preparations of the late-stage endotoxemic rats (Figure 4(a)). The HR in both early-stage and late-stage endotoxemia was not affected by Oro-A, but was significantly decreased by AVP. The rate of contraction of isolated atrial preparations from endotoxemic hearts also was significantly increased in the early-stage endotoxemia and remained increased until late-stage endotoxemia. These results are similar to those reported by others [29, 46, 47]. The rate of contractions in both stages was not affected by Oro-A or AVP, suggesting that both agents at the concentrations used do not directly affect these tissues or exhibit nonspecific effects. Again, the decreased HR by AVP only in the constant-pressure heart preparations but not in the constant-flow heart preparations suggests the involvement of indirect mechanism or secondary to decreased CF via vasoconstriction. This latter suggestion is likely, since Oro-A and AVP did not affect the contractile force of depressed atrial and ventricular strips. In this regard, Oro-A may not be beneficial for cardiogenic shock due to mechanical dysfunction of the heart.

Sepsis is a systemic inflammatory response of endotoxemia. In severe sepsis and septic shock patients with depressed cardiac function may have higher mortality than those without cardiac dysfunction [5]. Although the LPS-treated animal model may have its limitations in representing sepsis in human, it, however, can be useful to help determination of possible pathophysiology of endotoxemia [48]. Likewise, isolated hearts in Langendorff preparations are widely used to study mechanisms of myocardial functions in health and disease [49]. Our present findings, therefore, provide interesting information indicating that Oro-A improves, while AVP worsens the cardiac functions of endotoxemic rats. Although examination of effects of these two agents in a more clinically relevant model is needed, it is reasonable to suggest, based on the present animal studies, that Oro-A is a potentially favorable candidate for managing endotoxemic patients associated with cardiac dysfunction. For this same group of patients, however, use of AVP should be cautious.

It should be noted that our preliminary studies demonstrated that Oro-A post-treatment significantly reversed the LPS-induced systemic hypotension to normal ranges with significantly increased survival rate of the endotoxemic animals. Also, isolated mesenteric and tail arteries from LPS-induced septic rats constricted exclusively upon application of Oro-A. These results suggested that Oro-A did not further aggravate hypotension in endotoxemic shock. In this regard, coronary vessels seem to react differently from other systemic resistant vessels in response to Oro-A. The exact mechanism for the interesting and important difference remains to be fully determined. 

In summary, results of the present study indicate that Oro-A exerts a protective action with positive inotropy on isolated endotoxemic hearts via improved CF. In contrast, AVP further aggravates LPS-induced negative inotropy with decreased CF. Oro-A is an interesting candidate for providing not only a therapeutic strategy in treating endotoxemia or severe sepsis complicated with cardiac dysfunction, but also a new insight in understanding the pathophysiology of the LPS-induced cardiac dysfunction.

## Figures and Tables

**Table 1:** Changes of body temperature and hemodynamic parameters in 5 hr and 16 hr post-LPS (10 mg/kg, i.p.) rats.

Preparations	Parameters	Control	5 hrs	16 hrs
Whole animals	*n*	22	23	18
	BT (°C)	36.9 ± 0.1	39.0 ± 0.1*	38.2 ± 0.1^∗#^
	MAP (mmHg)	111 ± 2.1	112 ± 2.2	72 ± 2.3^∗#^
	HR (bpm)	336 ± 9.9	435 ± 7.5*	454 ± 5.2*

Isolated heart (constant pressure)	*n*	6	6	6
	CF (mL/min)	13 ± 0.1	7.1 ± 0.5*	15.6 ± 0.7^∗#^
	LVDP (mmHg)	80.8 ± 4.0	42.9 ± 4.8*	45.2 ± 4.1*
	HR (bpm)	292.1 ± 6.2	277.5 ± 2.0	338.4 ± 8.6^∗#^
	RPP (mmHg·bpm)	23321 ± 755	10836 ± 912*	13528 ± 560*

Isolated heart (constant flow)	*n*	6	6	6
	CPP (mL/min)	94.1 ± 0.6	122.2 ± 3.6*	53.8 ± 3.0^∗#^
	LVDP (mmHg)	89.9 ± 3.1	45.0 ± 2.2*	41.8 ± 1.9*
	HR (bpm)	299.4 ± 7.1	317.5 ± 7.9	312.5 ± 8.9
	RPP (mmHg·bpm)	26837 ± 841	14181 ± 976*	12763 ± 596*

Isolated right atrium	*n*	5	6	6
	Force (mg/mg)	12.5 ± 1.0	8.0 ± 1.0*	6.2 ± 0.6*
	Rate (bpm)^1^	321.1 ± 6.8	348.1 ± 7.9*	355.4 ± 13.2*

Isolated right ventricle	*n*	5	5	N/P
	Force (mg/mg)	13.9 ± 2.4	6.3 ± 0.3*	N/P
	Rate (bpm)^2^	120.0 ± 0.0	120.0 ± 0.0	N/P

Values are mean ± SEM. *P < 0.05 versus control group, ^#^ *P* <0.05 versus 5 hr LPS group. ^1^The spontaneous beating rate of the atrium was considered as sinoatrial electrical activity. ^2^The ventricular rate was triggered by electric stimulation with a frequency of 2 Hz. BT (body temperature); MAP (mean arterial pressure); HR (heart rate); CF (coronary flow); CPP (coronary perfusion pressure); LVDP (left ventricular developed pressure); RPP (rate-pressure product); bpm (beats per min); N/P (not performed).
